# Phenolic and anthocyanin content characterization related to genetic diversity analysis of *Solanum tuberosum* subsp. *tuberosum* Chilotanum Group in southern Chile

**DOI:** 10.3389/fpls.2022.1045894

**Published:** 2023-01-10

**Authors:** Anita Behn, Carolina Lizana, Felipe Zapata, Alvaro Gonzalez, Marjorie Reyes-Díaz, Derie Fuentes

**Affiliations:** ^1^ Instituto de Producción y Sanidad Vegetal, Facultad de Ciencias Agrarias y Alimentarias, Universidad Austral de Chile, Valdivia, Chile; ^2^ Biocomputing and Applied Genetics, Center for Systems Biotechnology, Fraunhofer Chile Research Foundation, Santiago, Chile; ^3^ Departamento de Ciencias Químicas y Recursos Naturales, Facultad de Ingeniería y Ciencias, Universidad de La Frontera, Temuco, Chile; ^4^ Centro de Biotecnología de Sistemas, Facultad de Ciencias de la Vida, Universidad Andres Bello, Santiago, Chile

**Keywords:** SSR, native potatoes, anthocyanin, diversity analysis, total phenolic content, *Solanum tuberosum*, Chilotanum Group

## Abstract

The potato (*Solanum tuberosum* L) is one of the four most important crops worldwide in production and consumption. It originated from South America along the Andes, where six hotspots of diversity known as subcenters of origin are described from Venezuela to Chiloe Island in Chile, and where the greatest diversity of potatoes in the world is found. Today, the use of ancestral genetic resources has gained significant relevance, recovering and producing foods with a greater nutrient content and beneficial to human health. Therefore, native potatoes possess a set of characteristics with great potential for use in potato breeding guided primarily to produce better feed, especially potatoes of the *Chilotanum* Group that are easily crossed with conventional varieties. The primary objective of this study was to evaluate 290 accessions of *S. tuberosum* subsp *tuberosum* belonging to the Chilotanum Group using a set of molecular markers and correlate them to its phenotypic traits for future use in breeding programs. For this purpose, 290 accessions were analysed through 22 specific microsatellites described previously, correlating them with flesh and skin colour, total phenolic content, and anthocyanin content. A division into groups considering all the 290 accessions resulted in two clusters using STRUCTURE analysis and seven different genetic clusters using UPGMA. The latter exhibited common phenotypic characteristics as well as anthocyanin content, strongly supporting a correlation between phenotypic traits and the genetic fingerprint. These results will enable breeders to focus on the development of potatoes with high polyphenol and anthocyanin content.

## Introduction

The potato (*Solanum tuberosum*) is currently the fourth most important crop after rice, wheat, and corn. It plays a relevant role in global nutrition and for the sustainable development goals of the United Nations related to zero hunger and feed security (FAO, 2008; Ahmadu et al., 2021; FAOSTAT, 2022). The crop has become increasingly relevant due to population growth and the need to increase the supply of healthy food especially in developing regions such as Africa and Latin America. However, in developed countries, an important global trend in recent years is related to achieving a healthier diet for the population that allows curbing the deleterious effects of obesity and other metabolic diseases through the use and selection of food enriched with beneficial molecules such as antioxidants and anti-inflammatory agents. In this regard, the use of various ancestral genetic agro-resources has become increasingly important, both for direct use and for breeding programs of commercial varieties (Zhang et al., 2017).

Potatoes are a staple food and a relevant component of daily diet. Its high nutritional value and high yield potential (Koch et al., 2019) make this crop substantial even in a food shortage. Potatoes have been considered a highly nutritious contribution to human health. The resistant starch or slowly digestible starch of potatoes exerts a healthy impact under habitual consumption, and it is complemented with its high nutritional value through antioxidants, minerals, and vitamins, including potassium, folate, magnesium, and zinc (Lizana et al., 2021). The superior satiating effect of potato compared to that of rice and pasta in a mixed meal was found to be consistent with its lower energy density (Zhang et al., 2017), exerting a positive effect also for persons conscious of healthy alimentation. Moreover, colored native potatoes are especially rich in polyphenols and anthocyanins, with antioxidant properties, in a broad range that is generally associated with tuber pigmentation (Ah-Hen et al., 2012; Valiñas et al., 2017; Inostroza-Blancheteau et al., 2018).

Pigmented potatoes generally contain antioxidant compounds as phenols (Rasheed et al., 2022) that have a molecular structure characterised by the presence of one or more phenolic rings. Phenolic compounds protect plants against biotic stress caused by herbivores, insects, and pathogens and also against abiotic stress such as tissue damages caused by excessive UV radiation and free radicals (Friedman, 2006; Pourcel et al., 2007). These compounds are synthesised in large quantities as a product of the secondary metabolism of plants (Quiñones et al., 2012). Within phenols, there are flavonoid pigments known as anthocyanins, which are present in almost all plants. Within the phenolic acids, potatoes contain the following anthocyanins: petunidin, delphinidin, malvidin, pelargonidin, and peonidin glycosides. These pigments have a suitable chemical structure to function as antioxidants, donating hydrogen ions or electrons to free radicals, displacing them with their aromatic structure (Kuskoski et al., 2004; Dudek et al., 2022). Diets rich in antioxidants are associated with lower risk of incidence of diseases such as atherosclerotic heart disease, certain cancers, and macular degeneration (Hertog et al., 1993). Therefore, the anthocyanins contained in potatoes improve the nutritional value of the tuber (Hertog et al., 1993; Khalid et al., 2020), in addition to being a source of health-promoting antioxidants (Brown, 2005; Khalid et al., 2020). Anthocyanins are efficient scavengers of free radicals possessing anti-inflammatory and antimicrobial properties (Kang and Choung, 2016) and are associated with a reduced risk of developing cardiovascular disease, osteoporosis, and diabetes (Bazzano et al., 2003; Thompson et al., 2009; Speer et al., 2020). They also possess beneficial health properties such as anticancer, hepatoprotective, and antiviral properties (Bontempo et al., 2015; Calderón-Reyes et al., 2020; Fernández and Lizana, 2020; Khalid et al., 2020; Rasheed et al., 2022). The anticancer effects of anthocyanin extract compounds help in suppressing early and advanced cell proliferation and induce apoptosis in colon cancer (Bethke and Jansky, 2008), as well as in human leukemia cells (De Masi et al., 2020). Furthermore, the anthocyanins present in purple-fleshed potatoes decrease the postprandial glycemic response (Stushnoff et al., 2008). Anthocyanins have also been demonstrated to possess significant potential in attenuating oxidative stress in the skin caused by UVB radiation and inflammatory response (Zhu et al., 2010). To achieve the maximum health benefits in potato consumption by obtaining the maximum concentration of polyphenolic compounds, it is necessary to include the colours and the cooking method of potato cultivars. Moreover, the tuber skin as a by-product differs from other by-products because of the presence of interesting nutritional and pharmaceutical constituents (Schieber and Saldaña, 2009). The temperature related to the cooking method, as well as the presence of oxygen and light, plays an important role in maximizing the preservation of anthocyanins in the tuber for human diet (Sui et al., 2014). The tubers of Chilean potatoes also possess high concentrations of anthocyanins and total phenolic content (TPC), resulting in a wide variability of colours in the skin and flesh (Ah-Hen et al., 2012; Valiñas et al., 2017).

The International Potato Center (CIP) declared six potato hotspots of diversity along the Andes Mountains from Venezuela in the North to Chiloe Island in the South (de Haan et al., 2014). Native potatoes from the northern Andes are primarily *S. tuberosum* subsp. *andigena*, and those from southern Chile are principally the Chilotanum Group (*S. tuberosum* subsp. *tuberosum*). Chile is considered a subcenter of origin of this crop. The Chilean germplasm provides new material for researchers interested in the origin of cultivated potatoes and *S. tuberosum* in Chile (Contreras, 2008). The Potato Genebank at the Universidad Austral de Chile (UACh) possesses a collection of accessions from all over the country that exhibit significant genetic diversity, traits of agronomic and culinary interest, and different degrees of resistance to biotic and abiotic stresses (López et al., 2015). López et al. (2015) revealed the characterisation of native accessions of the Potato Genebank at the UACh in terms of pest resistance using known molecular markers for genotyping.

There has been extensive research to date on potatoes due to the narrow genetic diversity among the known cultivars and the urgency to broaden the genetic pool to enhance breeding programs. Potatoes might be found in different environments, and because of this plasticity, they exhibit a great potential to adapt to the future climatic change caused by global warming and water stress conditions. In addition, more sustainable cultivation methods must introduce more abiotic and biotic resistance genes from wild potatoes. The 22 simple sequence repeat (SSR) markers developed by Ghislain et al. (2004) may be the most used markers to evaluate the diversity of native potatoes. The SSRs were used at the CIP, GLKWS, the Potato Genebank at the UACh, and other potato research centers to characterise them genetically. Other researchers have also worked with SSR markers, such as Wang et al. (2019) who proposed the model-based structure analysis, discriminating the population into two major subgroups, which can be further subdivided into seven groups based on collection sites Berdugo-Cely et al. (2017) analysed 809 Andigenum group accessions from the CCC using 5968 SNPs to determine the genetic diversity and population structure of the Andigenum germplasm, as well as the usefulness of this collection to map qualitative traits across the potato genome, showing that the CCC can be subdivided into two major groups associated with their ploidy level, viz., Phureja (diploid) and Andigena (tetraploid). Tillault and Yevtushenko (2019) fingerprinted 20 potato varieties, including five new genotypes developed in Alberta, Canada, using 10 SSR markers selected for their high discriminatory power. In that study, STM0037, STM1016, and STM1104 were found to be the best SSR markers to detect genetic differences between potato varieties. In addition, Salimi et al. (2016) determined a number of alleles per locus, ranging from 2 (STM1049) to 9 (STM1104), respectively, with an average of 6.22 to 9 alleles per locus. UPGMA dendrogram indicated that American genotypes exhibit higher expected heterozygosity than European genotypes, concluding that SSRs are appropriate markers for evaluating genetic diversity within and among potatoes from different geographical regions. Antonova et al. (2020) found that a variety of potatoes from different countries were combined into mixed groups, probably indicating the intensive exchange of breeding material, using the set of alleles in the 14 examined SSR loci. Due to the increased number of the genotyped accessions, the resolving power of only these 14 SSR markers was not sufficient because of the low values of bootstrap coefficients. In contrast, Italian accessions were subclustered into two groups and were found to be genetically highly similar to the South American germplasm, a finding that was also supported by the morphological and chemical measurements affecting their principal qualitative traits (Palumbo et al., 2019). A large number of commercial varieties in Europe and the United States have their ancestors in the potato germplasm of southern Chile. Therefore, the aims of the present study were to genetically characterise accessions from the Potato Genebank at the UACh belonging to the Chilotanum Group (*S. tuberosum* subsp. *tuberosum*) using SSR markers, chemically in terms of TPC and anthocyanin content and phenotypically in terms of tuber coloration in the flesh and skin, and to determine the correlations between them.

## Materials and methods

### Germplasm management and phenotypic trait screening

The tubers of 290 accessions from the Potato Genebank at the UACh were screened in two seasons, according to the scale for potato pigmentation from the CIP characterization parameters (Gómez, 2000). The definition of primary and secondary coloration of skin and flesh of tubers was based on the percentage of each colour, where primary colour is the one that is present in more than 50% of the skin or flesh respectively.

All accessions were grown in 2015/2016 and 2016/2017 at the Austral Experimental Station of the UACh located in Valdivia (39°47′ LS, 73°14′ W, 19 m a.s.l.). Two rows (each 4 m long and separated by 0.75 m) per accession were planted. The plant density was 5.3 plants/m^2^, which were fertilised with N (100 kg/ha), P_2_O_5_ (180 kg/ha), and K_2_O (60 kg/ha) at the planting time and N (50 kg/ha) plus K_2_O (60 kg/ha) at ridging, according to previous soil analyses (data not shown). Insecticides and fungicides were used to control biotic damage, according to manufacturer’s recommendations. Sprinkler irrigation was implemented to satisfy water requirements of plots. The harvest of tubers was done by hand when leaves and stem were dead (BBCH scale 97), according to each potato genotype. A total of 10 potato tubers were randomly selected from each accession for morphological characterisation, as well as for TPC and anthocyanin content determination. In total, 100 g of fresh potatoes (skin and flesh) was obtained from 10 different tubers, sliced, and lyophilized for chemical analysis two weeks after the harvest.

### TPC and specific anthocyanin content measurements

Total polyphenols were determined by a colorimetric assay using the Folin–Ciocalteu reagent according to the modified method of Singleton and Rossi (1965). For extraction, 0.2 g of lyophilized powder (skin + flesh) was mixed with 2 ml of methanol (80% v/v) and vortexed for 1 min. Then, the extracts were filtered through Whatman no. 5 filter paper. For quantification, 100 μL of supernatant was mixed with 750 μL of Folin–Ciocalteu reagent for 1 min and incubated at 22°C for 90 min in a water bath. Absorbance was read at 750 nm, and the TPC was determined from the standard curve of gallic acid.

The anthocyanin content was determined as described previously (Abdel-Aal and Hucl, 1999) Samples were lyophilized, and 1 g of powder was homogenised in 25 ml of acidified ethanol (pH 1), incubated for 30 min at 50°C, and centrifuged at 5400 g for 15 min at 4°C. The absorbance of the supernatant was measured at 530 and 700 nm and results were expressed in ppm of cyanidin 3-glucoside (Cy3Glu).

Anthocyanin-specific metabolites were also quantified by HPLC-DAD in the flesh of different native potato accessions. The extracts were prepared as described by Ruhland and Day (2000) with modifications made by Ribera et al. (2010), with a flow rate of 1 ml min^−1^. The anthocyanin compounds were measured through the content of anthocyanidins (aglycone anthocyanins) using the method proposed by Nyman and Kumpulainen (2001). Delphinidin, malvidin, petunidin, cyanidin, and peonidin were used as standards (Sigma Chemical Co. St. Louis, MO) and quantified using the same HPLC method described earlier. Signals were detected at 320 nm with the mobile phase acidified water and acetonitrile by HPLC-DAD using a Kromasil reverse phase (RP)-18 column (250 * 4.6 mm) equipped with a photodiode and a DAD (Jasco MD 2015 Plus).

### DNA extraction and SSR amplification

Total DNA from the 290 accessions was obtained from leaf tissue using the PureLink Genomic DNA kit (ThermoFischer, USA) according to manufacturer’s instructions. The quality of each purification product was evaluated using the QuBit 3.0 fluorometer and Infinite F200/M200 NanoQuant spectrophotometer at wavelengths of 260 and 280 nm. Then, these purified DNA samples were used for allele amplification in a volume of 25 µL using the KAPA2G Robust HotStart ReadyMix PCR kit.

The sequence of primers for the 22 SSRs were obtained from a previous study (Ghislain et al., 2004). PCR was performed according to the following program: initial denaturation for 3 min at 95°C, 35 cycles of 15 s at 95°C, 30 s annealing temperature, 15 s at 72°C, and final extension of 3 min at 72°C. The final amplification product was detected by capillary electrophoresis using the Fragment Analyzer kit (Advance Analytical) and the small fragment resolution kit (1–500 bp) DNF-905-K1000. The size of each allele was determined using the PROSize 2.0 software.

### Kinship, diversity, and population structure analyses

A population genetic analysis was performed with STRUCTURE software (Falush et al., 2003) on the 290 tetraploid potato genotypes using a Bayesian clustering analysis. A burning period of 5000 and 50000 MCMC reps were used as parameters for the length of the simulation. Four repetitions for 6 Ks were performed. Structure Harvester (Earl and von Holdt, 2012) was used for the evaluation. CLUMPP (Jakobsson and Rosenberg, 2007) was used to consolidate the results.

The kinship analysis was performed using the alleles previously described by Ghislain et al. (2004) for the 290 accessions, assigning values of 1 or 0 for the presence or absence of each allele in a binary form (CVS sheet). The PIC was calculated according to the following equation described by Nei (1973):


$$PIC\;=\;1-\sum^{}\left(fi^{2}\right)$$

where *fi* is the frequency of the *i*th amplified allele over all individuals of the population (Nei, 1973). The PIC values range between 0 and 1.0, where values close to 1.0 represent a good discriminatory power for that locus.

Genetic similarity and cluster analyses were performed using the DendroUPGMA server (Garcia-Valle et al., 1999), and Dice similarity coefficients were calculated based on presence or absence of alleles using the UPGMA method (unweighted pair group method with arithmetic averages) (Sokal and Michener, 1958). The distance matrix based on Dice coefficients represents an agglomerative hierarchical clustering representation of mean genetic distances within each species. Each calculated matrix of Newick descriptors was built and used to construct a distance-based dendrogram using the SeaView tool (Gouy et al., 2010).

### Genetic fingerprint analysis

To identify the unique minimal allelic profile representing each potato accession, a full vector representation was constructed for all the 290 accessions, containing a complete binary allelic representation for each accession (**Supplementary Figure 1**). To evaluate the genetic fingerprint that represents a unique combination of SSRs for each accession, an algorithm was designed to search a relational database containing all binary allelic representations and the minimum and unique combinations of SSRs. Thus, these vector profiles would allow identifying a specific accession through a unique PCR band profile.

### Statistical analysis

Descriptive statistics was used to characterise the TPC and anthocyanin content of the genotypes of the Potato Genebank. All data concerning phenotype features such as the skin and flesh colour and spot distribution were grouped into seven genetic clusters. Their statistical significance was evaluated by two-way ANOVA and Tukey’s *post hoc* test to determine statistical differences between the clusters and features.

## Results

**Figure 1:**
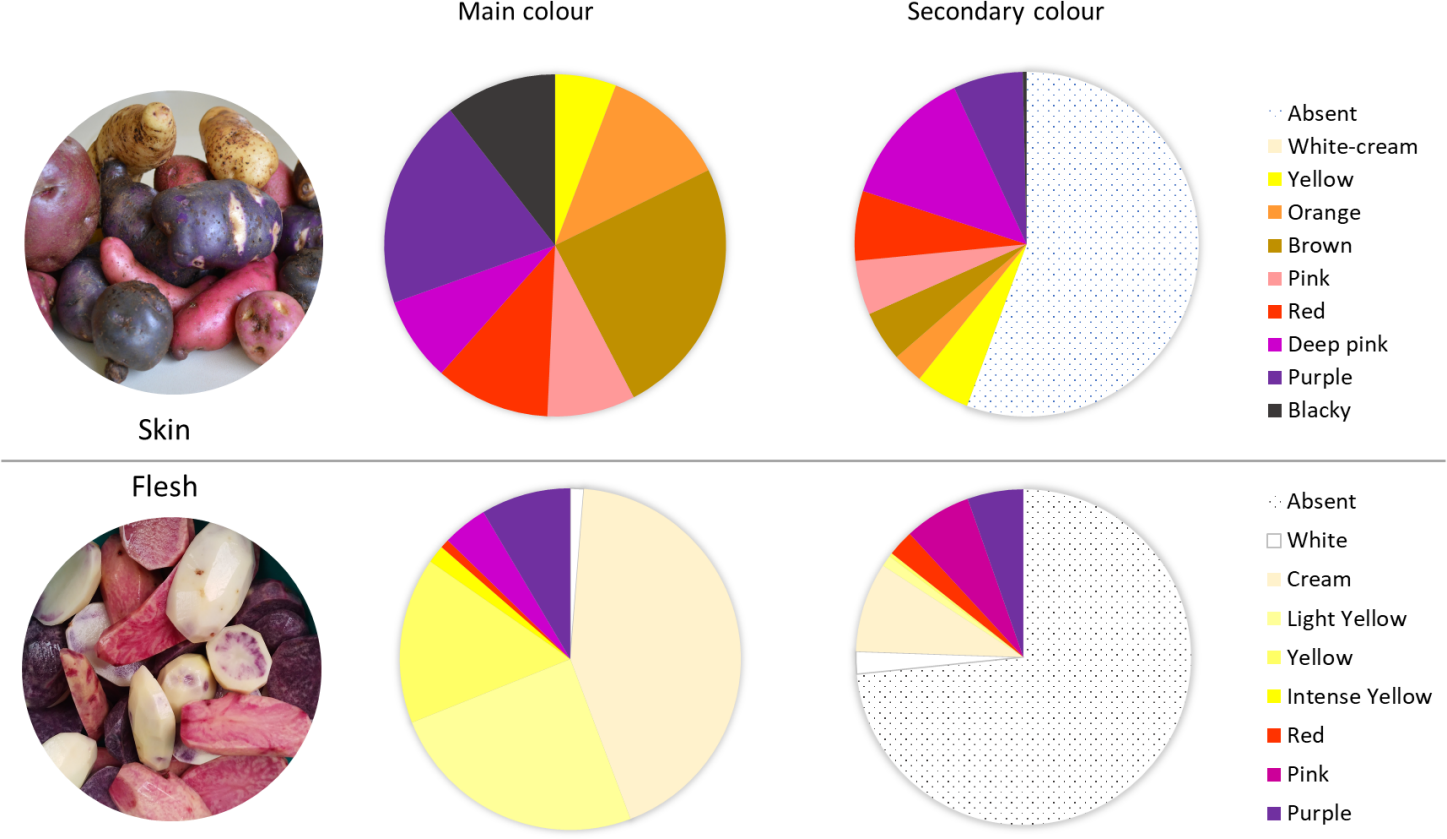
Characterization and distribution of accessions in terms of primary (main) or secondary color of the tuber skin (upper panel) and according to primary or secondary flesh color (lower panel).

The 290 native potato accessions belonging to the Potato Genebank at the UACh were cultivated and harvested, and their tubers were evaluated phenotypically using the standards defined by the CIP (Gómez, 2000; CIP, Perú). The traits were evaluated in 10 tubers per accession. Within the 290 accessions, cream (41%), yellow (26%), or white (2%) was the primary flesh color of the tubers, totaling 69% of the screened collection. Pigmented tubers primarily showed purple coloration, where 21 accessions had purple flesh, 10 had pink flesh, and only 1 accession showed a slightly red pigmentation in the flesh. Altogether, they represented 10% of total accessions, whereas 46 accessions showed the same tones as a secondary colour (16%). Tubers with secondary coloration showed white, cream, or yellow as the primary color and pink or violet as the secondary colour, marbled or with circular distribution in the vascular ring, presenting different color distributions in the flesh (**Figure 1**). In total, 78 accessions (26%) showed some anthocyanin-related colour.

Regarding the skin colours, 164 accessions (57%) exhibited some variation in skin pigmentation from pink to blackish, and 83 accessions showed the same color range as the secondary colour (29%), totaling up to 85% of total accessions, revealing a greater abundance of anthocyanins and phenols in the skin than in the flesh of the screened potatoes. A total of 30 accessions (10%) showed blackish colour and 56 accessions showed purple pigmentation (19%) in the skin. Regarding the secondary colour, only 2 accessions exhibited marbled distribution with a blackish pigmentation, 19 accessions showed a purple coloration, and 30 accessions showed a pink coloration. The most frequent primary colour of the skin was yellow (24%), and 59% of the screened accessions showed no secondary colour (**Figure 1**).

### Total phenolic content

**Figure 2:**
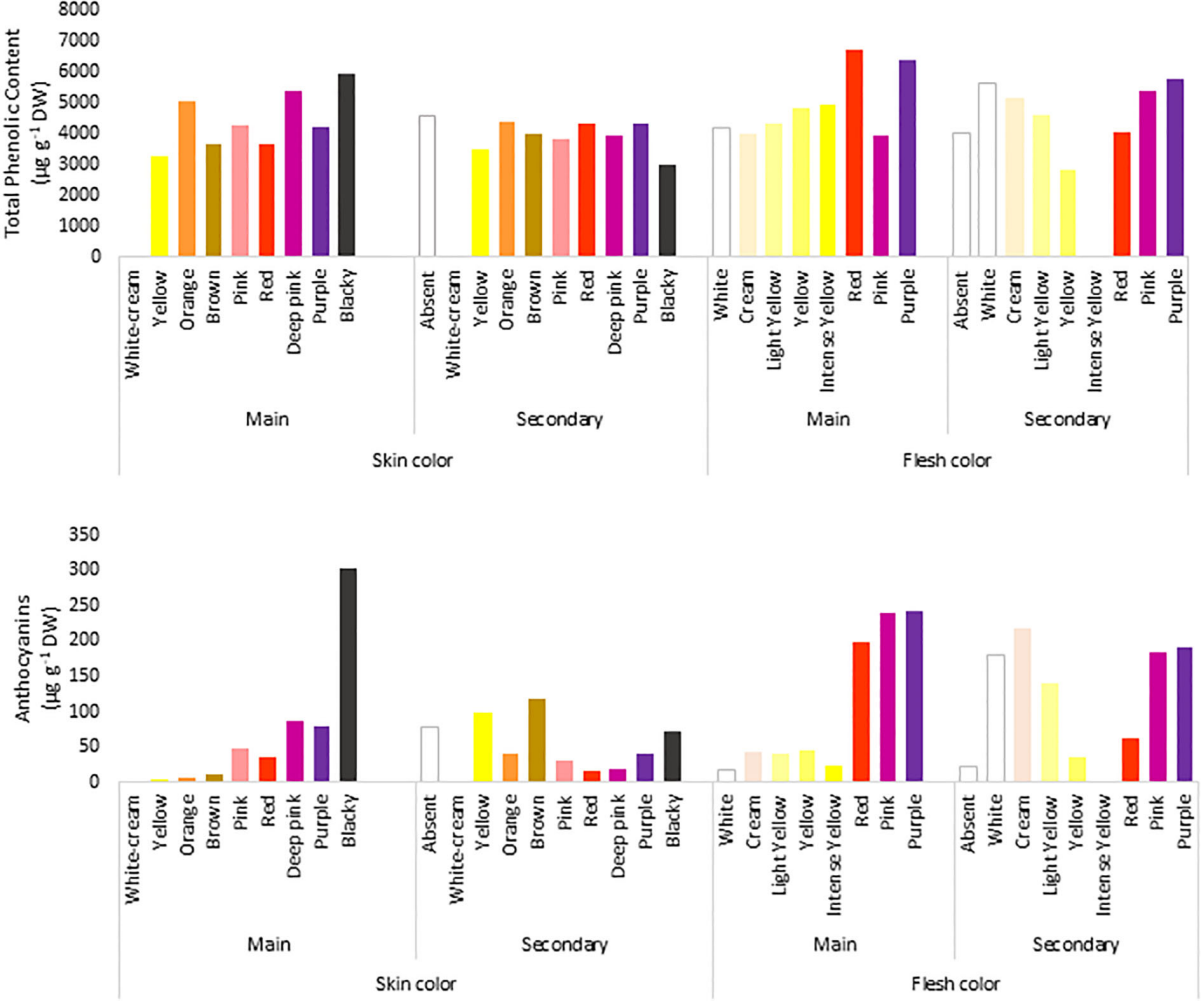
Concentration of total phenolic compounds (µg Gallic acid/g DW) determined by Folin–Ciocalteu method (upper panel) and total anthocyanin content (µg/g of cyanidin 3-glucoside) were extracted in ethanol acidified using Abdel-Aal and Hucl (1999) method (lower panel) in the 290 accessions of the Potato GenBank at the UACh.

The TPC of the accessions ranged from 1070 to 18,103 µg g^−1^ DW, with a mean of 4286 µg g^−1^ DW and a median of 3787 µg g^−1^ DW, and 39% of the genotypes had higher TPC values than the average value (**Figure 2A**). Moreover, 98% of the potato accessions had<10,000 µg g^−1^ DW, and only 6 accessions exceeded that value. Within the total number of accessions, there were two highly recognizable native genotypes with purple flesh and skin, whose TPC values were >15,000 µg g^−1^ DW. In general, potatoes with low TPC had white flesh and brown skin, and those with high TPC had purple flesh and blackish skin, showing an 18-fold difference in TPC between them.

To evaluate the relationship between the colour and TPC of tubers, the genotypes were grouped according to their skin and flesh colour and the average TPC per group was calculated according to each colour category (**Figure 2A**). In terms of flesh colour level, the analyses showed that genotypes with purple flesh as the primary colour had 1.6 times more TPC of up to 6383 µg g^−1^ DW than genotypes with pink (3937 µg g^−1^ DW) and cream (3999 µg g^−1^ DW) flesh (**Figure 2A**). Because of the difference in the proportion of genotypes with purple and pink marbled flesh when the accessions were compared, the tubers in those accessions with purple flesh as the secondary colour showed slightly less TPC of 5792 µg g^−1^ DW than the tubers with pink marbled flesh whose TPC reached 5386 µg g^−1^ DW.

### Anthocyanin content

The anthocyanin content in the tubers showed a broader range of variation than that of TPC, ranging from 0.3 to 920 µg g^−1^ DW. The average concentration was only 61.3 µg g^−1^ DW, and the median was 17.7 µg g^−1^ DW. In total, 80% of all genotypes had anthocyanin content below the average, and only 10 genotypes exceeded 400 µg g^−1^ DW. Three of them showed anthocyanin content between 600 and 700 µg g^−1^ DW, whereas one genotype showed the highest content of 920 µg g^−1^ DW. Potato tubers with less anthocyanin content had cream flesh and brown skin (0.3 µg g^−1^ DW), and those with high anthocyanin content had purple flesh and blackish skin (920 µg g^−1^ DW), showing a 3066-fold difference in the content between them.

**Figure 3:**
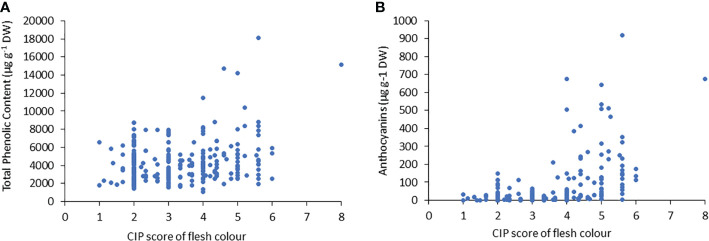
Correlation between **(A)** the total phenolic content (TPC) and **(B)** anthocyanin content, related to tuber flesh colors with the CIP score.

Similar to TPC, the genotypes with purple flesh as the primary color had the highest concentration of anthocyanins, and those with pink flesh had 1.2 times more anthocyanin content than tubers with red flesh (**Figure 2B**). The highest concentrations of anthocyanins were found in accessions with flesh colours ranging from red to purple, which were 7-fold higher than those found in tubers with flesh color ranging white to intense yellow (**Figure 2B**). The TPC and anthocyanin contents were higher in accessions with higher flesh scores than in accessions with red, pink, and purple flesh colours (**Figure 3**).

**Figure 4:**
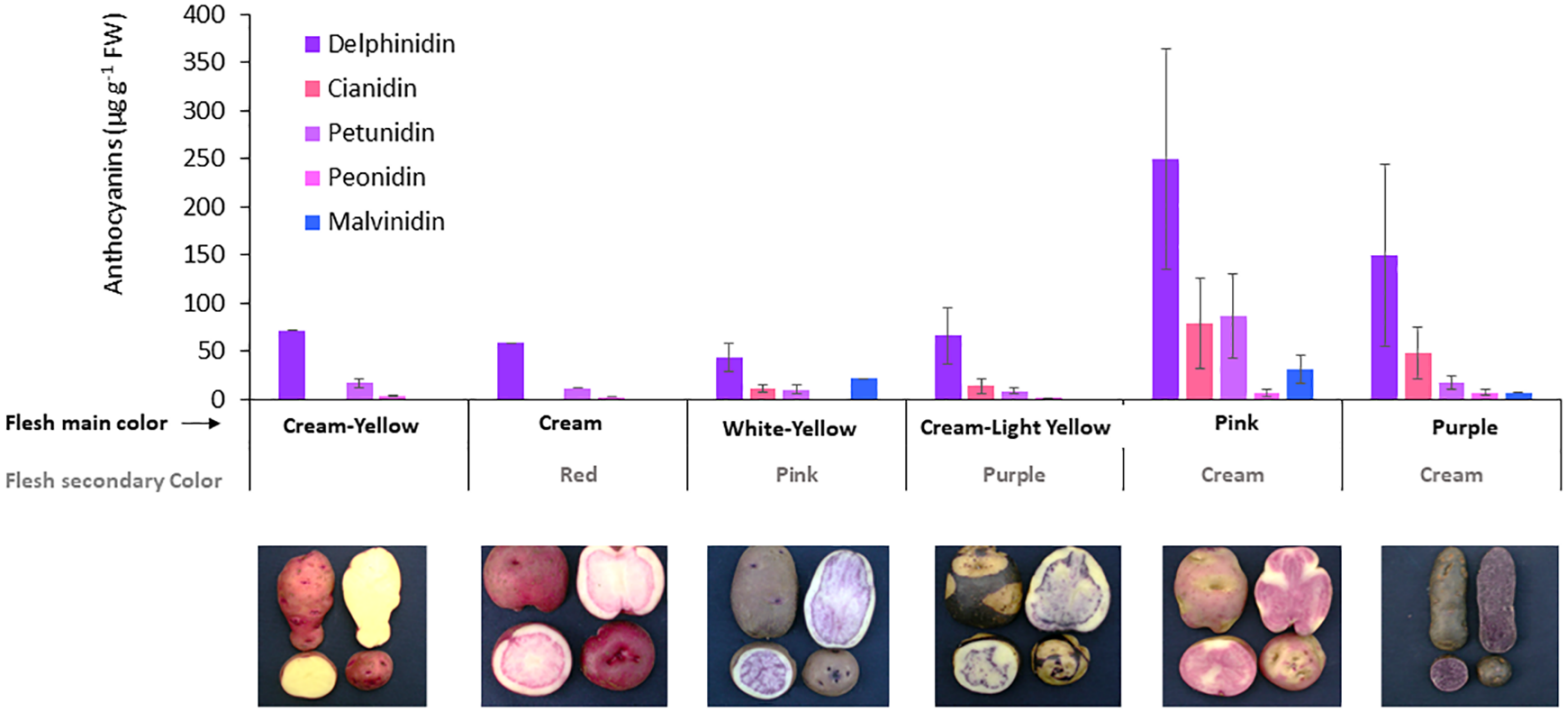
Anthocyanin profile in tubers for accession groups with different flesh color. The number of accessions for each group represented in the figure is cream-yellow = 2; cream/red = 1; white-yellow/pink = 6; cream-light yellow/purple = 4; pink/cream = 7; and purple/cream = 5. The X axis shows the combinations of primary and secondary colors in the flesh according to how accessions were grouped. Images show one accession representative of each group.

The specific anthocyanins were analysed by HPLC-DAD in the whole of potato tubers, which revealed higher concentrations of delphinidin of up to 553 µg g^-1^ DW, followed by cyanidin (2–205 µg g^−1^ DW) and petunidin (2–236 µg g^−1^DW), and lower concentrations of peonidin (1–18 µg g^−1^ DW) and malvidin (7–67 µg g^-1^ DW). Delphinidin was the most frequent anthocyanin found in the screened potato tubers, closely followed by cyanidin and petunidin; these anthocyanins were related to purple, magenta, and dark red flesh colours, respectively. All anthocyanins, except malvidin, showed a significant relationship (p< 0.01) between their specific concentrations and the total anthocyanin content of each accession (**Figure 4**). Specific anthocyanins were associated with different flesh colours of tubers as the primary or secondary colour (**Figure 4**). Higher concentrations and a more diverse profile of anthocyanins were detected in accessions with pink or purple flesh as the primary colour, supporting the relationship between the more intense colour and the higher anthocyanin content.

Regarding the anthocyanin profile obtained for each accession, there were differences between pink and purple accessions, where both the anthocyanin concentration and the proportion of predominant anthocyanin type differed (**Figure 4**).

### Genetic analysis

Regarding the genotypic characterization, the genetic relationship among all the 290 accessions was evaluated based on the presence or absence of SSRs within the potato genomes described by Ghislain et al. (2004). The number of alleles per SSR ranged from 2 to 13 with an average of 7.36 alleles per SSR, and the PIC ranged from 0.43 to 0.91 with an average of 0.77, considering that the highest value was 1. The discriminatory power of the 22 SSR markers is represented in **Table 1**, which includes the PIC values calculated for each SSR. The most discriminatory SSRs corresponded to STM3012 and STM3023a with a PIC value of 0.91, followed by STM0037 (PIC value of 0.89) and the following three SSRs with a PIC value of 0.87: STM1104, STM0030, and STM1053. The largest number of alleles per SSR was observed in STM3023a, STM0037, and STM0030, and the lowest number of alleles per SSR was observed in STM1017 and STM1049 (2 and 3, respectively). STM1106 and STM2013 did not amplify any product in any reaction and were thus eliminated from further calculations.

**Table 1:** SSRs, number of alleles, and PIC.

Locus	Size	Number of alleles	Alleles	Polymorphism information content (PIC)
STM1049	190–203	3	190 ± 2, 200 ± 2, 203	0.58
STM2022	188–217	4	188 ± 2, 205 ± 2, 245 ± 2, 217 ± 2	0.68
STM1053	172–184	5	172 ± 1, 175 ± 1, 178 ± 1, 181 ± 1, 184 ± 1	0.87
STM3023a	180–204	13	180+1, 182 + 1, 184 + 1, 186 + 1, 188 + 1, 190 + 1, 192 + 1, 194 + 1, 196 + 1, 198 + 1, 200 + 1, 202 + 1, 204 + 1	0.91
STM1031	271–293	4	271 ± 1, 274 ± 1, 288 ± 1 or 285 ± 1, 291 ± 1 or 293	0.73
STPoAc58	237–261	6	237 ± 2, 251 ± 1, 245 ± 1, 258 ± 1, 261 ± 1, 254 ± 1	0.74
STM0019a	146–235	8	190 ± 2, 196 ± 2 or 199, 204 ± 1 or 207 ± 2, 211 ± 1 or 213 ± 1, 232 ± 1 or 235 ± 1, 146 ± 1, 171 ± 1, 157	0.84
STM0031	172–197	4	172 ± 1, 190 ± 1, 197 ± 1, 183 ± 1	0.74
STM1052	212–267	7	214 ± 1 or 212, 222 ± 1 or 220, 230 ± 1 or 228 ± 1, 242 ± 1, 255 ± 1 or 253, 259 ± 1 or 257, 267 ± 1	0.83
STM1104	168–188	8	168 ± 1, 171 ± 1, 174 ± 1, 179 ± 1, 182 ± 1, 188 ± 1, 177 + 1, 185 ± 1	0.87
STM1016	235–267	9	247 ± 1, 252 ± 2, 257 ± 1, 260 ± 1, 244 ± 1, 235 ± 2, 267 ± 2, 263 ± 1, 242	0.84
STGBSS	125–145	7	125 ± 2, 131 ± 1, 134 ± 1, 137 ± 1, 140 or 142, 145 ± 1, 128 or 129	0.83
STWAX-2	218–249	9	218 ± 1, 224 ± 1 or 222, 228 ± 1, 231 ± 1, 234 ± 1, 239 ± 1 or 237, 243 ± 1 or 241, 243 ± 1 or 241, 214 ± 1, 249	0.81
STM3012	168–215	8	168 ± 1, 172 ± 2, 199 ± 1, 202 ± 1, 205 ± 1, 215 ± 1, 212 ± 1, 196 ± 1	0.91
STM0037	76–96	10	78+1, 80 + 1, 84 + 1, 86 + 1, 88, 91 + 1, 93 + 1, 96, 82 + 1, 76 + 1	0.89
STM0030	111–164	11	111, 119 + 1, 124, 135, 139 + 1, 141, 143, 146 + 1, 157, 160, 164	0.87
STM2030	180–270	4	180 ± 3, 210 ± 3, 248 ± 3, 270 ± 3	0.66
STM1064	194–201	4	194 ± 1, 197 ± 1, 199 + 1, 201 + 1	0.65
STM1058	114–129	5	120 ± 1, 123 ± 1, 126 ± 1, 129 ± 1, 114 ± 1	0.78
STM1017	135–139	2	135 ± 2, 139 ± 1	0.43

### Hierarchical structure or genotype clusters

**Figure 5:**
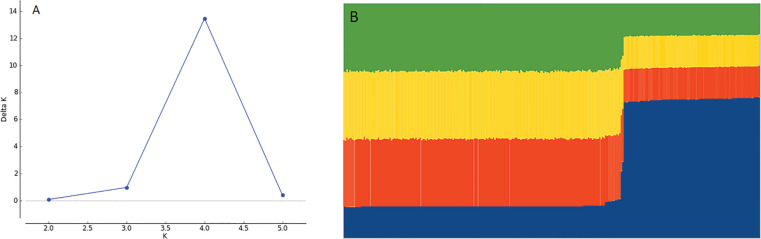
**(A)** Plot for Delta K at different K values. Higher value at K = 4 with Delta K = 14. **(B)** Bar plot of STRUCTURE assuming K = 4 subpopulations showing 2 clusters of genotypes.

The genetic analysis performed using STRUCTURE and Structure Harvester software with 4 repetitions for 6 Ks, showed that K=4 was the best value for K (**Figure 5A**). CLUMPP was used to consolidate the results of the 4 repetitions for K=4 and the result was plotted into a bar plot for the STRUCTURE simulation with K=4 subgroups, showing that genotypes formed two different clusters (**Figure 5B**), where cluster 1 comprises 195 accessions, while cluster 2 contains 95 accessions.

**Figure 6:**
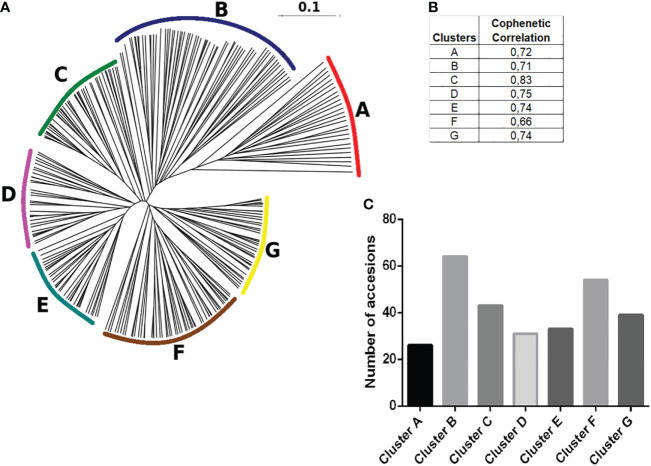
Hierarchical clustering of varieties. **(A)** The DendroUPGMA server was used to calculate the Dice similarity matrix coefficients between accessions using the UPGMA method to calculate an agglomerative hierarchical clustering representation of mean genetic relatedness within each species represented in a dendrogram with regard to allelic profiles contained within the 22 SSRs. **(B)** Cophenetic correlation coefficients of UPGMA clusters, values close to 1 represent the usefulness of the clustering method, and values between 0.6 and 1 are those with the highest correlation. **(C)** Total number of accessions distributed in the different genetic subgroups determined in this study.

**Figure 7:**
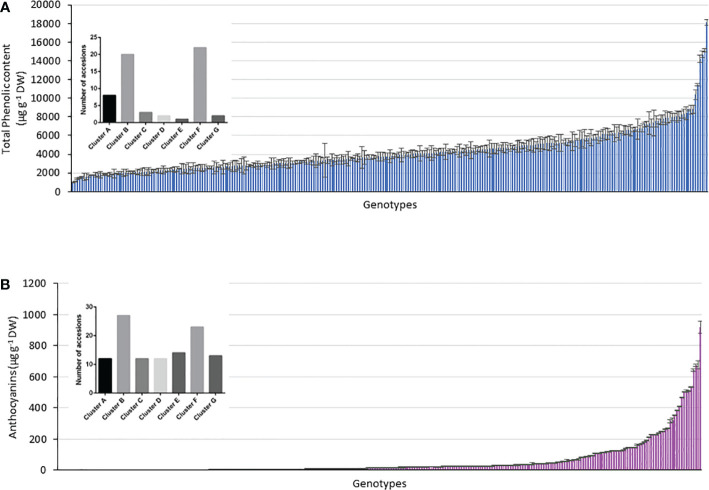
Relationship between genotype and phenotype within the accessions considering the genotypes above the respective mean values in **(A)** TPC, **(B)** anthocyanin content and the 290 accessions assigned to clusters A–G.

A distance matrix based on Dice coefficients was generated using the 290 native potato accessions in terms of SSR binary fingerprints. Neighbor joining clustering analyses separated them into seven hierarchical groups (clusters), which were arbitrarily named from A to G, and established the genetic kinship or relationship between all of them (**Figure 6A**). The cophenetic correlation coefficient for each cluster ranged from 0.66 to 0.82, indicating the usefulness of the clustering method, where values close to 1 represented a perfect match (**Figure 6B**). As shown in **Figure 7C**, clusters B and F had the highest number of genotypes of 64 and 54, respectively. **Supplementary Table 1** presents thedetailed information about the accessions belonging to each cluster.

Using the kinship grouping among the 290 accessions and considering that a major objective was to identify possible genetic markers that differentiate potato accessions with higher TPC and anthocyanin content, we selected those accessions whose TPC and anthocyanin content were above the mean value. **Figure 7** shows the histograms of the TPC and anthocyanin content of all the analyzed accessions, including their mean values (anthocyanins = 61.3 µg g^-1^ DW and TPC = 4286 µg g^-1^ DW). Above each histogram, the genetic clusters are shown, considering the accessions with values above the mean in each case. Thus, 58 accessions showed a higher value than the mean anthocyanin content (61.4 µg g^-1^ DW), which were distributed as follows among the clusters: 8 accessions in cluster A, 20 accessions in cluster B, 3 accessions in cluster C, 2 accessions in cluster D, 1 accession in cluster E, 22 accessions in cluster F, and 2 accessions in cluster G. However, 113 accessions showed values of >4286 µg g^-1^ DW (mean TPC of the population), which were distributed as follows: 12 accessions in cluster A, 27 accessions in cluster B, 12 accessions in cluster C, 12 accessions in cluster D, 14 accessions in cluster E, 23 accessions in cluster F, and 13 accessions in cluster G (**Figures 7A, B**). The black bars in **Figure 7** indicate those clusters with a significant difference in the accessions with high content (p< 0.05). In this manner, clusters B and F included the largest number of accessions with high TPC and anthocyanin content (**Figures 7A, B**). Interestingly, cluster C included accessions with high TPC and anthocyanin content. However, clusters E and D agglomerated accessions with low anthocyanin content (**Figure 7B**).

Based on the same correlation analysis, we determined the correlation between the tuber genotype and phenotype, considering the primary and secondary colours of the skin and flesh. These colours were classified into nine categories (white cream, yellow, orange, brown, pink, red, deep pink, purple, and blackish) for skin and eight categories (white, cream, light yellow, yellow, intense yellow, red, pink, and purple) for flesh, according to an adapted classification proposed by the CIP, Perú. Considering all groups, the occurrence of these phenotypic traits was evaluated, and the probability of the occurrence of any of these traits in each cluster was quantified. Furthermore, the statistical significance weight for each probability was evaluated to distinguish it as a characteristic trait of the cluster. In relation to the primary skin color, there were no strong correlation between characteristics and clusters (**Supplementary Table 2**). However, for the secondary colour, cluster F showed a high probability of grouping accessions with secondary deep pink colour (**Supplementary Table 2**, p< 0.05). Regarding flesh colour, clusters B, C and F showed a high probability to agglomerate accessions with cream, and cluster G the accessions with light yellow flesh (p< 0.05; **Supplementary Table 2**). Furthermore, regarding the secondary flesh colour, cluster A showed a high probability to agglomerate accessions with pink flesh, (p< 0.05), cluster G agglomerate red flesh potatoes and cluster F showed a high probability to agglomerate accessions with cream flesh (p< 0.05; **Supplementary Table 2**).

### Genetic fingerprints

To determine a strategy for differentiating potato accessions by genetic fingerprinting, it is essential to calculate the minimal number of SSR markers required to identify with a unique PCR pattern in one potato accession. Considering the phenotype in the cluster analysis, 113 accessions with TPC above the mean value and 58 accessions with anthocyanin content above the mean value were evaluated. Clusters B and F contained the maximum number of accessions from the total number of accessions, and within the groups, cluster B contained 47% and cluster F contained 59% of accessions with enhanced TPC and anthocyanin content, respectively.

Regarding the number of SSRs present in the seven clusters, STM1016 that was present in all clusters (A–G) and STM3023a, STM1104, STM1016, and STGBSS were polymorphic in a large number of genotypes in clusters A, B, and F. These clusters are important because they have high potential to include genotypes with high levels of anthocyanins and pink-like potato genotypes. These findings suggest that the presence of these SSRs is important in the expression of these phenotypes and should be considered in further investigations.

## Discussion

Due to the narrow genetic diversity among the known potato cultivars and the need to broaden the genetic pool to enhance breeding programs, there has been extensive research on potatoes. A growing interest in functional foods has stimulated investigation on pigmented potatoes for their potential effects on human health related to their abundance of phenolic compounds and anthocyanins, as well as a plant protection effect. The biological activities of polyphenols in potatoes might be helpful for breeders in designing new varieties with numerous health benefits, for both pharmaceutical and nutraceutical industries (Rasheed et al., 2022). The inclusion of anthocyanins as a characteristic target in breeding programs can ensure the development of cultivars to satisfy the nutritional requirements in human consumption in the developing world (Mattoo et al., 2022).

The high genetic and phenotypic diversity within the Potato Genebank at the UACh contains the native potato diversity of the subcenter of origin of the *S. tuberosum* subsp. *tuberosum* Chilotanum Group (Spooner et al., 2007). Contreras and Castro (2008) compiled the major source of phenotypic data on the number and diversity of native varieties by UPOV characterization. The present study has complemented the phenotypic information by exploring the diversity in colors, phenols, and anthocyanins, as well as their interactions, and their genetic kinship relationship using molecular markers (SSR), with the aim to elucidate key components required for increased content of nutritional components for human feed and contribute to global food security.

Flesh-colored potatoes can represent an additional source of bioactive compounds, particularly acylated anthocyanins, in the human diet (Bellumori et al., 2017). Anthocyanins play a vital role because of their potential health benefits, and therefore, elucidating their biosynthesis has become a research focus, and one of the most investigated pathways in plants. The antioxidant intake in humans is focused on TPC and anthocyanin content, which are present in higher concentrations in potato tubers with red, pink, and purple flesh. In our study, there was a positive correlation among the primary color of the flesh, TPC, and anthocyanin content. TPC showed a lower correlation with flesh pigmentation (0.23) than with anthocyanin content (0.44). However, the highest anthocyanin concentrations were found in accessions with flesh colors ranging from red to purple, which were 7-fold higher than those found in tubers with flesh colors ranging from white to intense yellow (**Figure 2B**). The correlation coefficient between TPC and anthocyanin content was 0.5, showing correlation between the scores of the different genotypes, which are consistent with published data in different plants (Liu, 2013; Diep et al., 2020). The measurements of TPC and anthocyanin content performed on skin and flesh together were also consistent with those reported by Ah-Hen et al. (2012) and Ru et al. (2019) who observed that TPC was much higher in coloured potato tubers than in white, cream, or yellow potato tubers. Ru et al. (2019) found 3- to 4-fold higher TPC in red or purple potatoes than in white or yellow potatoes. Ah-Hen et al. (2012) found 10 times higher TPC in a purple accession than in a white potato tuber. That is consistent with the present investigation, where 16-fold higher TPC was detected in a purple potato accession when compared with white potato tubers. The mean TPC values of all purple flesh accessions were only 1.5-fold higher than those of pink or cream flesh accessions. The anthocyanin content showed no differences between the purple and pink tubers, but it was 5.8-fold higher than that in tubers with cream flesh. Chilean native potatoes with the highest TPC and anthocyanin content possess mostly blackish skin and/or purple flesh, indicating the traditional accessions with blackish skin and dark purple flesh, and these results are consistent with those reported by Ah-Hen et al. (2012). These data are also consistent with those of other studies, which reported that colored potatoes have high concentrations of anthocyanins, reaching 1200 mg kg^-1^ FW (Ruiz et al., 2018; Ercoli et al., 2021; Alarcón et al., 2022).

Colored potatoes had higher TPC and higher antioxidant capacity than potatoes with yellow and white flesh (Ru et al., 2019). In our study, a couple of genotypes were characterized as having purple flesh, but with low TPC, as shown by the mean TPC of 6187 µg g^−1^ DW in potatoes with purple flesh, which varied between the highest content of 18,103 µg g^−1^ DW and the lowest content of 1937 µg g^−1^ DW. The same trend was observed for anthocyanin content in potatoes with purple flesh. These results were not consistent with the results reported by Ru et al. (2019).

In contrast, Ru et al. (2019) observed pelargonidin in potatoes with red flesh, as well as pelargonidin and petunidin in potatoes with purple flesh, wherein the latest presenting a higher total anthocyanin content than potatoes with red flesh. However, in the present study, purple tubers did not necessarily possess higher mean anthocyanin content than pink tubers. Moreover, purple Chilean potatoes with the highest TPC and anthocyanin content contain the highest concentration of delphinidin (829 µg g^−1^ DW) but no petunidin. However, the highest score of petunidin also corresponds to a purple tuber, concluding that both anthocyanins could provide purple color to potato tubers. When a subset of accessions of the Potato Genebank at the UACh was examined using molecular markers of three anthocyanin synthesis genes, a greater allelic diversity was observed at the P locus that is related to the synthesis of blue/purple petunidin-based anthocyanins, were alleles correlated positively with purple pigmentation in most of the accessions (Solís et al., 2022), that is consistent with the CIP scoring in tubers of the Potato Genebank, as most coloured potatoes scored corresponded to purple primary or secondary color. It should be emphasized that the presence of a higher delphinidin content in the screened potato accessions makes them an interesting source of bioactive compounds for human health. The features of delphinidin include 1) its colour that appears as a blue-reddish or purple pigment in plants, 2) stability under acidic conditions, 3) patented for several therapeutic effects, and 4) the maximum inhibitory effect on lipid peroxidation and ${\text{O}}_{2}^{\cdot -}$ scavenging activity compared with other anthocyanidins (Khoo et al., 2017; Calderón-Reyes et al., 2020; Xu et al., 2020).

As the present results were based on the analysis of raw potatoes, they might change according to the cooking method, as they demonstrated negative effects on the contents of vitamin C, total phenolics, phenolic acids, and DPPH radical-scavenging activity (Fang et al., 2022). Currently, it is reported that cooked potatoes have decreased total anthocyanin concentrations by approximately 3%–59%. Despite these decreases, the potato genotypes had high levels of total phenols as well as high levels of antioxidant activity; hence, even with the drastic process of cooking, these results are remarkable contributors to the antioxidant activity of potato genotypes (Ercoli et al., 2021).

To investigate the genetics and its possible relationship with TPC and anthocyanin content, we performed the kinship analysis of the 290 genotypes and correlated the phenotypic and genotypic data. As the accessions were planted and harvested at the same time in the same field, as well as under the same growing conditions and cultivation techniques, we assumed that the variation between the accessions in terms of tuber color, TPC, and anthocyanin content can be attributed only to the genotypes.

The heterozygous autotetraploid *S. tuberosum* ssp. *tuberosum*, with four homologous sets of chromosomes (2n = 4x = 48), exhibited several heterogeneous genotypes and phenotypes. Even the selection of genotypes for crossing is primarily dependent on morphological traits, and genetic relatedness measured based on molecular markers help predict parental performance and improve heterotic effects. The 22 SSR markers developed by Ghislain et al. (2004) may be the most used markers to evaluate the diversity of native potatoes, for example at the CIP, GLKWS, the Potato Genebank at the UACh, and other potato research centers. The approach used in this investigation based on the 22 SSR markers (Ghislain et al., 2004) enables merging the data with the named genebanks around the world. It could also be useful for the study of the belonging to different spatiotemporal groups, as done by Spanoghe et al. (2022) by examining a substantial panel of 1219 potato varieties using 35 microsatellite markers (SSR) to evaluate the genetic diversity. Ghislain et al. (2009) developed a potato genetic identification kit to differentiate 93.5% and 98.8% of 742 landraces using 24 and 51 SSR markers, respectively, indicating that SSR markers could efficiently identify potato germplasm at the genetic level. Hence, a high genotypic diversity might be found in the present study because the 290 accessions were different and part of a wide pool in the collection of *S. tuberosum* Chilotanum Group in the Potato Genebank. As the allelic diversity or the mean number of alleles per locus measures the genetic variation, a comparison was made with previous investigations on native potatoes. The mean number of alleles per locus was 7.4, similar to that observed by Muñoz et al. (2016) who reported a mean of 8.0 using only four SSRs, but it was less than that reported by Solano et al. (2013) who showed a mean of 9.16 alleles per locus using seven SSRs. In total, 162 alleles were found, including 20 SSRs, as STM1106 and STM2013 showed no amplification. The PIC of the different SSRs in the present study ranged broadly from 0.43 to 0.91. The mean value (0.77) was comparable with the PIC reported in different investigations that explored the genetic diversity of potato accessions, for example Muñoz et al. (2016) who reported PIC values ranging from 0.77 to 0.86.

While STRUCTURE analysis cluster into two groups with K=4, the hierarchical analysis (UPGMA dendrogram) showed 7 cluster. The reviewing of the accession’s distribution between the two approaches showed that the vast majority (> 90%) of the accessions belonging to subgroups A, B, D and F identified through UPGMA analysis were also grouped together in the first STRUCTURE cluster, while the accessions grouped in clusters E and G were mostly (>80%) grouped in the second STRUCTURE cluster. Only the accessions belonging cluster C showed a more equal distribution (55%-45%) among both STRUCTURE clusters. These results suggest that the genetic analysis approach did not resolve the degree of relatedness between the 290 accessions, and the genetic structure in the genebank, which will require further analysis. Notwithstanding the above, the simple observation of the diversity of phenotypes represented in shapes, colours, TPC and anthocyanin content in the genebank hints the high genetic variations that had not been possible to address so far. The result of the STRUCTURE analysis (**Figure 5B**) showed the need of a more complex and in-depth analysis of the accessions of the Potato Genebank. Selga et al. (2022) suggested that breeding material and the studied Genebank collection were closely related, showing a low degree of population structure between the groups. That result agree with the present results, as only 4 subgroups separated in two clusters were determined on that analysis.

The distribution of accessions in 7 different subgroups (UMPGA analysis, **Figure 6A**), made sense when correlating the phenotypic differences between them. Duan et al. (2019) developed a UPGMA dendrogram based on 20 polymorphic SSR markers for Chinese breeding lines, indicating that all the 217 cultivars were closely related and lacked the formation of distinct clusters; in contrast to the present kinship analysis that showed the formation of groups clustering all the 290 accessions into seven different genetic clusters, which indicated a number of common phenotypic characteristics as anthocyanin contents, strongly supporting a correlation between the phenotypic traits and the genetic fingerprint, accounting for the high genetic diversity in the Potato Genebank. Considering the SSRs present in the seven clusters with high cophenetic scores, STM1016 was predominant because of its presence in all clusters (A–G), and also the markers STM3023a, STM1104, and STGBSS as they were polymorphic in a large number of genotypes in clusters A, B, and F, appearing to have an importance in the expression of these phenotypes. These clusters are important because they have high potential to accumulate genotypes with high levels of anthocyanins and pink-like potatoes. Muñoz et al. (2016) selected STM1016 among the four SSR markers used that were sufficiently informative to identify 320 different allelic phenotypes and thus 320 potential varieties. They also used STM1106, the SSR that did not amplify in the present study, which was considered to be not diagnostic for the Chilotanum group. Tillault and Yevtushenko (2019) fingerprinted 20 potato varieties, including 5 new genotypes developed in Alberta, Canada, using 10 SSR markers, wherein the number of alleles per locus ranged from two for the SSR marker STPoAc58 to six for STM0030 and STM0037. When compared with the present results, STM0030, STM0037, and STM3023a had the highest number of alleles of 11, 10, and 13 per locus, respectively. Tillault and Yevtushenko (2019) described the markers STM0037, STM1016, and STM1104 as the best SSR markers to detect genetic differences between potato varieties, which is broadly consistent with the present investigation. Another research on potatoes screened a collection of 264 Russet and non-Russet breeding clones and varieties through fingerprinting using 23 SSR markers, resulting in 142 polymorphic alleles. The number of alleles produced per SSR varied from 2 to 10, with an average of 6.2 alleles per marker. The PIC of SSRs ranged from 0.37 to 0.89 with an average of 0.77 (Bali et al., 2018). Despite the narrow-expected diversity in the investigation done by Bali et al., 2018, the scores were similar to those found in the present study, with the number of alleles varying from 2 to 13, and the PIC ranging from 0.43 to 0.91 with the same average of 0.77. However, Jian et al. (2017) detected 190 alleles on 20 SSR loci, and all the SSR alleles were polymorphic among these potato germplasms with an average of 9.5 alleles per SSR locus, ranging from 2 to 23, showing a larger number of alleles than that of the Potato Genebank.

Salimi et al. (2016) used SSRs to determine allelic diversity within and among potatoes from different geographical regions and reported numerous alleles per locus, ranging from two (STM1049) to nine (STM1104), respectively, which is consistent with the present data, where STM1049 showed three alleles and STM1104 showed eight alleles. Antonova et al. (2020) investigated the genetic diversity of potatoes using the set of alleles in the 14 examined SSR loci but indicated that with an increased number of the genotyped accessions, the resolving power of only these 14 SSR markers was not sufficient because of the low values of bootstrap coefficients. Duan et al. (2019) used 16 parental cultivars widely used in breeding to screen 138 SSR markers, where 20 were polymorphic that were used to analyze the genetic diversity of 217 potato cultivars grown in China. Based on the PIC values and the clarity of PCR amplification bands, 11 SSR markers were selected that could differentiate all the 217 cultivars. The 22 SSRs used in the present investigation were diagnostic and sufficient to evaluate genetic diversity and cluster the genotypes in groups. Wang et al. (2019) successfully discriminated the population into two major subgroups using SSRs, which can be further subdivided into seven groups based on collection sites. In the present investigation, seven groups were found, but they have not yet been correlated to the collection site.

Although SSRs are widely used to evaluate genetic diversity in potatoes throughout the world, Campos and Ortiz (2020) described that single nucleotide polymorphisms (SNPs) are increasingly used predominantly due to recent advances in genome sequencing technology, abundance of SNPs in most crop plants, reduced labor required to collect data, and price per data point. Furthermore, Parra-Galindo et al. (2021) found a genomic region in chromosome 10 that harbored SNPs with the strongest association with anthocyanin content in GWAS and underlined the existence of pleiotropic genes or anthocyanin biosynthesis clusters. Seven QTLs were identified to be involved in the genetic control of the anthocyanin content in cooked tubers. These QTLs explained from 31.3% to 44.4% of the phenotypic variance (anthocyanidin content and composition) (erratum: Parra-Galindo et al., 2020; Parra-Galindo et al., 2021). However, Campos and Ortiz (2020) mentioned that it is often challenging to identify SNP markers in polyploids such as the potato, due to separating allelic versus homologous SNPs or determining dosage in autopolyploid, both of which increase the rate of false positives. In the present study, we first decided to work with SSRs, because most analyses conducted to date on genetic diversity in potato genebanks have used microsatellites. Moreover, we achieved our primary objective of correlating the phenotypic and genotypic scores and identifying clusters B and F containing accessions with high amounts of TPC and anthocyanin content and a couple of molecular markers that well described both relevant clusters.

## Conclusion

The narrow genetic diversity observed among modern potato varieties necessitates the need for investigations like this one so that potato breeders can enhance genetic diversity in parental clones. The native potatoes of the Chilotanum Group (*S. tuberosum* subsp. *tuberosum*) can be crossed with the common potato varieties and present a source of resistance to disease and stress, as well as high contents of polyphenols and anthocyanins, which make them contributors to breeding for designing new varieties with health benefits to satisfy the nutritional requirements in human consumption and for plant health.

The coloured accessions of the Potato Genebank primarily possessed purple and pink flesh as secondary colors and less than as primary colours. Within the 290 accessions, the primary color of the tubers was cream (41%), yellow (26%), or white (2%), and 10% of the total accessions showed purple, pink, or red flesh, whereas 46 accessions showed the same tone as the secondary color (16%). In total, 78 accessions had some anthocyanin-related colour, corresponding to 26% of total accessions. Regarding the skin colour, 164 accessions (57%) showed some variation in skin pigmentation. ranging from pink to blackish.

The TPC and anthocyanin content showed significant variation, observing genotypes with higher contents than some fruits known for their antioxidant capacity. The genotypes showing purple flesh as the primary color had the highest concentrations of anthocyanins; those with purple flesh and blackish skin had the highest TPC of 18,103 µg g^−1^ DW and the highest anthocyanin content of 920 µg g^−1^ DW. All anthocyanins, except malvidin, showed a significant relationship (p< 0.01) between their specific concentrations and the total anthocyanin content of each accession. Similarly, delphinidin was the most frequent specific anthocyanin found in the screened potato tubers, which was different from the published data on the anthocyanins of purple potatoes, indicating that it is a potentially interesting compound for further investigation.

Genetic diversity was evaluated using SSRs, resulting in 146 alleles with an average of 7.36 alleles per SSR and a mean PIC of 0.77; these data were consistent with the published information on other native genotypes. The most discriminatory SSRs corresponded to STM3012 and STM3023a with a PIC value of 0.91, and the largest number of alleles per SSR was observed in STM3023a, STM 0037, and STM 0030.

The STRUCTURE analysis using K=4, formed two different clusters, with 195 and 95 accessions, respectively. The seven clusters obtained using the UPGMA dendrogram showed high cophenetic coefficients. The correlation of genotypes with TPC and anthocyanin content greater than the mean value indicated that clusters B (47%) and F (59%) contained the largest number of genotypes with a high content in each case. Cluster F and A had a high probability of grouping accessions with secondary deep pink colour of the skin, concluding that the clustering does present a correlation to colours, TPC, and anthocyanin. The possible SSR that differentiated the potato accessions with higher TPC and anthocyanin content was STM1016 that was present in all clusters (A–G), and also STM3023a, STM1104, STM1016, and STGBSS, the markers that were polymorphic in a large number of genotypes in clusters A, B, and F. Finally, the high variability in tuber colors, TPC, and anthocyanin content was consistent with the high genetic diversity screened using genotype-specific SSR markers, which support the use of this genetic material in potato breeding.

## Data availability statement

The original contributions presented in the study are included in the article/**Supplementary Material**. Further inquiries can be directed to the corresponding authors.

## Author contributions

AB and CL did the phenotypic screening and chemical analysis, MR-D did the anthocyanins analyses per HPLC. FZ and DF performed the SSR amplifications on the DNA accessions and AG developed the kinship analysis and then the association between the different groups of accessions and their anthocyanin and phenol content. All authors contributed to the article and approved the submitted version.

## References

[B1] Abdel-Aal E. S. Hucl P. (1999). A rapid method for quantifying total anthocyanins in blue aleurone and purple pericarp wheats. Cereal Chem. 76, 350–354. doi: 10.1094/CCHEM.1999.76.3.350

[B2] Ah-Hen K. Fuenzalida C. Hess S. Contreras A. Vega-Gálvez A. Lemus-Mondaca R. (2012). Antioxidant capacity and total phenolic compounds of twelve selected potato landrace clones grown in southern Chile. Chil. J. Agric. Res. 72, 3–9. doi: 10.4067/S0718-58392012000100001

[B3] Ahmadu T. Abdullahi A. Ahmad K. (2021). The role of crop protection in sustainable potato (Solanum tuberosum l.) production to alleviate global starvation problem: An overview. in m. yildiz and y. ozgen (Eds.), solanum tuberosum - a promising crop for starvation problem. IntechOpen 354. doi: 10.5772/intechopen.100058

[B4] Alarcón S. Tereucán G. Cornejo P. Contreras B. Ruiz A. (2022). Metabolic and antioxidant effects of inoculation with arbuscular mycorrhizal fungi in crops of flesh-coloured solanum tuberosum treated with fungicides. J. Sci. Food Agric. 102, 2270–2280. doi: 10.1002/jsfa.11565 34625964

[B5] Antonova O. Klimenko N. S. Rybakov D. A. Fomina N. A. Zheltova V. V. Novikova L.. (2020). SSR analysis of modern Russian potato varieties using DNA samples of nomenclatural standards. Plant Biotechnol. Breed. 3, 77–96. doi: 10.30901/2658-6266-2020-4-o2

[B6] Bali S. Patel G. Novy R. Vining K. Brown C. Holm D.. (2018). Evaluation of genetic diversity among russet potato clones and varieties from breeding programs across the united states. PloS One 13, e0201415. doi: 10.1371/journal.pone.0201415 30067845 PMC6070254

[B7] Bazzano L. A. Serdula M. K. Liu S. (2003). Dietary intake of fruits and vegetables and risk of cardiovascular disease. Curr. Atheroscler. Rep. 5, 492–499. doi: 10.1007/s11883-003-0040-z 14525683

[B8] Bellumori M. Innocenti M. Michelozzi M. Cerretani L. Mulinacci N. (2017). Coloured-fleshed potatoes after boiling: Promising sources of known antioxidant compounds. J. Food Compos. Anal. 59, 1–7. doi: 10.1016/j.jfca.2017.02.004

[B9] Berdugo-Cely J. Valbuena R. I. Sánchez-Betancourt E. Barrero L. S. Yockteng R.. (2017). Genetic diversity and association mapping in the Colombian Central Collection of Solanum tuberosum L. Andigenum group using SNPs markers. PloS One 12 (3), e0173039. doi: 10.1371/journal.pone.0173039 28257509 PMC5336250

[B10] Bethke P. C. Jansky S. H. (2008). The effects of boiling and leaching on the content of potassium and other minerals in potatoes. J. Food Sci. 73, H80–H85. doi: 10.1111/j.1750-3841.2008.00782.x 18576999

[B11] Bontempo P. De Masi L. Carafa V. Rigano D. Scisciola L. Iside C.. (2015). Anticancer activities of anthocyanin extract from genotyped solanum tuberosum l. “Vitelotte”. J. Funct. Foods. 19, 584–593. doi: 10.1016/j.jff.2015.09.063

[B12] Brown C. R. (2005). Antioxidants in potato. Am. J. Potato Res. 82, 163–172. doi: 10.1007/BF02853654

[B13] Calderón-Reyes C. Silva Pezoa R. Leal P. Ribera-Fonseca A. Cáceres C. Riquelme I.. (2020). Anthocyanin-rich extracts of calafate (Berberis microphylla g. forst.) fruits decrease *in vitro* viability and migration of human gastric and gallbladder cancer cell lines. J. Soil Sci. Plant Nutr. 20, 1891–1903. doi: 10.1007/s42729-020-00260-8

[B14] Campos H. Ortiz O. (2020). The potato crop: its agricultural, nutritional and social contribution to humankind (Springer Nature) 518 (Cap4), 128. Available at: https://link.springer.com/content/pdf/10.1007/978-3-030-28683-5.pdf?pdf=button

[B15] Contreras A. (2008). Uso de especies silvestres y cultivadas en el mejoramiento de la papa. Agrosur 36, 115–129. doi: 10.4206/agrosur.2008.v36n3-01

[B16] Contreras A. Castro I. (2008). Catálogo de variedades de papas nativas de Chile (Chile: Universidad Austral de Chile), 234. Available at: www.potatogenebank.cl.

[B17] de Haan S. Polreich S. Juarez H. Rodriguez F. Ccanto R. Alvarez C.. (2014). “The chirapaq Ñan initiative: Establishment of a long-term on-farm monitoring network for potato landrace diversity,” in Book of abstracts. international conference on enhanced genepool utilization capturing wild relative and landrace diversity for crop improvement. Cambridge (UK). Eds. Dias S. Dulloo E. Maxted N. Kell S. Thorn E. Smith L. Preston J. Hutchinson S. (Rome (Italy: Bioversity International), 28.

[B18] De Masi L. Bontempo P. Rigano D. Stiuso P. Carafa V. Nebbioso A.. (2020). Comparative phytochemical characterization, genetic profile, and antiproliferative activity of polyphenol-rich extracts from pigmented tubers of different solanum tuberosum varieties. Molecules 25, 233. doi: 10.3390/molecules25010233 31935970 PMC6983029

[B19] Diep T. Pook C. Yoo M. (2020). Phenolic and anthocyanin compounds and antioxidant activity of tamarillo (Solanum betaceum cav.). Antioxid. (Basel) 9, 169. doi: 10.3390/antiox9020169 PMC7070485 32085645

[B20] Duan Y. Liu J. Xu J. Bian C. Duan S. Pang W.. (2019). DNA Fingerprinting and genetic diversity analysis with simple sequence repeat markers of 217 potato cultivars (Solanum tuberosum l.) in China. Am. J. Potato Res. 96, 21–32. doi: 10.1007/s12230-018-9685-6

[B21] Dudek A. Spiegel M. Strugała-Danak P. Gabrielska J. (2022). Analytical and theoretical studies of antioxidant properties of chosen anthocyanins; a structure-dependent relationships. Int. J. Mol. Sci. 23, 5432. doi: 10.3390/ijms23105432 35628243 PMC9141991

[B22] Earl D. A. von Holdt B. M. (2012). STRUCTURE HARVESTER: a website and program for visualizing STRUCTURE output and implementing the evanno method. Conserv. Genet. Resour. 4 (2), 359–361. doi: 10.1007/s12686-011-9548-7

[B23] Ercoli S. Parada J. Bustamante L. Hermosín-Gutiérrez I. Contreras B. Cornejo P.. (2021). Noticeable quantities of functional compounds and antioxidant activities remain after cooking of colored fleshed potatoes native from southern Chile. Molecules 26, 314. doi: 10.3390/molecules26020314 33435441 PMC7827549

[B24] Falush D. Stephens M. Pritchard J. K. (2003). Inference of population structure using multilocus genotype data: linked loci and correlated allele frequencies. Genetics 164 (4), 1567–1587. doi: 10.1093/genetics/164.4.1567 12930761 PMC1462648

[B25] Fang H. Yin X. He J. Xin S. Zhang H. Ye X.. (2022). Cooking methods affected the phytochemicals and antioxidant activities of potato from different varieties. Food Chem. 14, 100339. doi: 10.1016/j.fochx.2022.100339 PMC9133768 35634223

[B26] FAO (2008). Año internacional de la papa 2008: Nueva luz sobre un tesoro enterrado. reseña de fin de año. Available at: https://www.fao.org/3/i0500s/i0500s.pdf

[B27] FAOSTAT (2022) Producción de cultivos. Available at: http://www.fao.org/faostat/es/#data/QC.

[B28] Fernández R. Lizana X. C. (2020). Antocianinas en Solanum tuberosum: Una revisión. Agrosur 48 (2), 1–8. doi: 10.4206/agrosur.2020.v48n2-01

[B29] Friedman M. (2006). Potato glycoalkaloids and metabolites: Roles in the plant and in the diet. J. Agric. Food Chem. 54, 8655–8681. doi: 10.1021/jf061471t 17090106

[B30] Garcia-Valle S. Palau J. Romeu A. (1999). Horizontal gene transfer in glycosyl hydrolases inferred from codon usage in escherichia coli and bacillus subtilis. Mol. Biol. Evol. 16, 1125–1134. doi: 10.1093/oxfordjournals.molbev.a026203 10486968

[B31] Ghislain M. Núñez J. Herrera M. Pignataro J. Guzman F. Bonierbale M.. (2009). Robust and highly informative microsatellite-based genetic identity kit for potato. Mol. Breed. 23, 377–388. doi: 10.1007/s11032-008-9240-0

[B32] Ghislain M. Spooner D. M. Rodriguez F. Villamón F. Núñez J. Vásquez C.. (2004). Selection of highly informative and user-friendly microsatellites (SSRs) for genotyping of cultivated potato. Theor. Appl. Genet. 108, 881–890. doi: 10.1007/s00122-003-1494-7 14647900

[B33] Gómez R. (2000). Guía para las caracterizaciones morfológicas básicas en colecciones de papas nativas. centro internacional de la papa (CIP), germoplasma de papa, dpto. de mejoramiento y recursos genéticos (Lima, Perú: CIP).

[B34] Gouy M. Guindon S. Gascuel O. (2010). SeaView version 4: A multiplatform graphical user interface for sequence alignment and phylogenetic tree building. Mol. Biol. Evol. 27, 221–224. doi: 10.1093/molbev/msp259 19854763

[B35] Hertog M. G. Feskens E. J. Kromhout D. Hollman P. C. H. Katan M. B. (1993). Dietary antioxidant flavonoids and risk of coronary heart disease: the zutphen elderly study. Lancet 342, 1007–1011. doi: 10.1016/0140-6736(93)92876-u 8105262

[B36] Inostroza-Blancheteau C. de Oliveira Silva F. M. Durán F. Solano J. Obata T. Machado M.. (2018). Metabolic diversity in tuber tissues of native chiloé potatoes and commercial cultivars of solanum tuberosum ssp. tuberosum l. Metabolomics 14, 138. doi: 10.1007/s11306-018-1428-7 30830417

[B37] Jakobsson M. Rosenberg N. A. (2007). CLUMPP: a cluster matching and permutation program for dealing with label switching and multimodality in analysis of population structure. Bioinformatics 23 (14), 1801–1806. doi: 10.1093/bioinformatics/btm233 17485429

[B38] Jian W. Lu H. Wang R. Y. He M. M. Liu Q. C. (2017). Genetic diversity and population structure of 288 potato (Solanum tuberosum l.) germplasms revealed by SSR and AFLP markers. J. Integr. Agric. 16, 2434–2443. doi: 10.3390/plants10040752

[B39] Kang M. A. Choung S. Y. (2016). Solanum tuberosum l. cv hongyoung extract inhibits 2, 4-dinitrochlorobenzene-induced atopic dermatitis in NC/Nga mice. Mol. Med. Rep. 14, 3093–3103. doi: 10.3892/mmr.2016.5595 27510042 PMC5042769

[B40] Khalid W. Khalid M. Z. Aziz A. Tariq A. Ikram A. Rehan M.. (2020). Nutritional composition and health benefits of potato: A review. Adv. Food Nutr. Sci. 5, 7–16. doi: 10.21065/AdvFoodNutriSci.5.7

[B41] Khoo H. E. Azlan A. Tang S. T. Lim S. M. (2017). Anthocyanidins and anthocyanins: colored pigments as food, pharmaceutical ingredients, and the potential health benefits. Food Nutr. Res. 61, 1361779. doi: 10.1080/16546628.2017.1361779 28970777 PMC5613902

[B42] Koch M. Naumann M. Pawelzik E. (2019). Cracking and fracture properties of potato (Solanum tuberosum l.) tubers and their relation to dry matter, starch, and mineral distribution. J. Sci. Food Agric. 99, 3149–3156. doi: 10.1002/jsfa.9530 30548622

[B43] Kuskoski E. M. Asuero A. G. García-Parilla M. C. Troncoso A. M. Fett R. (2004). Actividad antioxidante de pigmentos antociánicos. Ciênc. Tecnol. Aliment. 24, 691–693. doi: 10.1590/S0101-20612004000400036

[B44] Liu R. (2013). Health-promoting components of fruits and vegetables in the diet. Adv. Nutr. 4, 384S–392S. doi: 10.3945/an.112.003517 23674808 PMC3650511

[B45] Lizana X. Sandaña P. Behn A. Ávila-Valdés A. Ramírez D. Soratto R.. (2021). “Potato,” in Crop physiology case histories for major crops, 1st Edition. Eds. Sadras V. Calderini D. (London. Reino Unido: Academic Press is an imprint of Elsevier), 551–588.

[B46] López M. Riegel R. Lizana X. Behn A. (2015). Identification of viruses and nematodes resistance genes in the chilotanum potato genebank of the universidad austral de Chile. Chil. J. Agric. Res. (JAR) 75, 320–327. doi: 10.4067/S0718-58392015000400008

[B47] Mattoo A. K. Dwivedi S. L. Dutt S. Singh B. Garg M. Ortiz R. (2022). Anthocyanin-rich vegetables for human consumption–focus on potato, sweetpotato and tomato. Int. J. Mol. Sci. 23, 2634. doi: 10.3390/ijms23052634 35269776 PMC8910313

[B48] Muñoz M. Folch C. Rodriguez F. Kalazich J. Orena S. Santos J.. (2016). Genotype number and allelic diversity overview in the national collection of Chilean potatoes. Potato Res. 59, 227–240. doi: 10.1007/s11540-016-9329-5

[B49] Nei M. (1973). Analysis of gene diversity in subdivided populations. Proc. Natl. Acad. Sci. U. S. A. 70, 3321–3323. doi: 10.1073/pnas.70.12.3321 4519626 PMC427228

[B50] Nyman N. A. Kumpulainen J. T. (2001). Determination of anthocyanidins in berries and red wine by high-performance liquid chromatography. J. Agric. Food Chem. 49, 4183–4187. doi: 10.1021/jf010572i 11559107

[B51] Palumbo F. Galvao A. C. Nicoletto C. Sambo P. Barcaccia G. (2019). Diversity analysis of sweet potato genetic resources using morphological and qualitative traits and molecular markers. Genes 10, 840. doi: 10.3390/genes10110840 31653056 PMC6895877

[B52] Parra-Galindo M. A. Piñeros-Niño C. Soto-Sedano J.C. Mosquera-Vasquez T. (2020). Chromosomes I and X harbor consistent genetic factors associated with the anthocyanin variation in potato agronomy 2019, 9, 366. Agronomy 10, 532. doi: 10.3390/agronomy10040532

[B53] Parra-Galindo M. A. Soto-Sedano J. C. Mosquera-Vasquez T. Roda F. (2021). Pathway based analysis of anthocyanin diversity in diploid potato. PloS One 16 (4), e0250861. doi: 10.1371/journal.pone.0250861 33914830 PMC8084248

[B54] Pourcel L. Routaboul J. M. Cheynier V. Lepiniec L. Debeaujon I. (2007). Flavonoid oxidation in plants: from biochemical properties to physiological functions. Trends Plant Sci. 12, 29–36. doi: 10.1016/j.tplants.2006.11.006 17161643

[B55] Quiñones M. Miguel M. Aleixandre A. (2012). Los Polifenoles, compuestos de origen natural con efectos saludables sobre el sistema cardiovascular. Nutr. Hosp. 27, 76–89. doi: 10.3305/nh.2012.27.1.5418 22566306

[B56] Rasheed H. Ahmad D. Bao J. (2022). Genetic diversity and health properties of polyphenols in potato. Antioxidants 11, 603. doi: 10.3390/antiox11040603 35453288 PMC9030900

[B57] Ribera A. E. Reyes-Díaz M. Alberdi M. Zuñiga G. E. Mora M. L. (2010). Antioxidant compounds in skin and pulp of fruits change among genotypes and maturity stages on highbush blueberry (Vaccinium corymbosum l.) grown in southern Chile. J. Soil Sci. Plant Nutr. 10, 509–536. doi: 10.4067/S0718-95162010000200010

[B58] Ruhland C. T. Day T. A. (2000). Effects of ultraviolet-b radiation on leaf elongation, production and phenylpropanoid concentrations in deschampsia antarctica and colobanthus quitensis in Antarctica. Physiol. Plant 109, 244–251. doi: 10.1034/j.1399-3054.2000.100304.x

[B59] Ruiz A. Aguilera A. Ercoli S. Parada J. Winterhalter P. Contreras B.. (2018). Effect of the frying process on the composition of hydroxycinnamic acid derivatives and antioxidant activity in flesh colored potatoes. Food Chem. 268, 577–584. doi: 10.1016/j.foodchem.2018.06.116 30064800

[B60] Ru W. Pang Y. Gan Y. Liu Q. Bao J. (2019). Phenolic compounds and antioxidant activities of potato cultivars with white, yellow, red and purple flesh. Antioxidants 8, 419. doi: 10.3390/antiox8100419 31547004 PMC6827044

[B61] Salimi H. Bahar M. Mirlohi A. Talebi M. (2016). Assessment of the genetic diversity among potato cultivars from different geographical areas using the genomic and EST microsatellites. Iran. J. Biotechnol. 14, 270. doi: 10.15171/ijb.1280 28959345 PMC5434997

[B62] Schieber A. Saldaña M. (2009). Potato peels: A source of nutritionally and pharmacologically interesting compounds–a review. Food 3, 23–29. doi: 10.7939/R33T9DM0H

[B63] Selga C. Chrominski P. Carlson-Nilsson U. Andersson M. Chawade A. Ortiz R. (2022). Diversity and population structure of Nordic potato cultivars and breeding clones. BMC Plant Biol. 22, 350. doi: 10.1186/s12870-022-03726-2 35850617 PMC9290215

[B64] Singleton V. L. Rossi J. A. (1965). Colorimetry of total phenolics with phosphomolybdic-phosphotungstic acid reagents. Am. J. Enol. Vitic. 16, 144–158.

[B65] Sokal R. R. Michener C. D. (1958). A statistical method for evaluating systematic relationships. Univ. Kans. Sci. Bull. 38, 1409–1438.

[B66] Solano J. Mathias M. Esnault F. Brabant P. (2013). Genetic diversity among native varieties and commercial cultivars of solanum tuberosum ssp. tuberosum l. present in chile. electron. J. Biotechnol. 16, 6. doi: 10.2225/vol16-issue6-fulltext-15

[B67] Solís J. L. Muth J. Canales J. Lizana C. Pruefer D. Riegel R.. (2022). Allelic diversity of three anthocyanin synthesis genes in accessions of native solanum tuberosum l. ssp. tuberosum at the potato genebank of the universidad austral de Chile. Genet. Resour. Crop Evol. 69, 297–314. doi: 10.1007/s10722-021-01230-4

[B68] Spanoghe M. Marique T. Nirsha A. Esnault F. Lanterbecq D. (2022). Genetic diversity trends in the cultivated potato: a spatiotemporal overview. Biology 11, 604. doi: 10.3390/biology11040604 35453803 PMC9026384

[B69] Speer H. D’Cunha N. M. Alexopoulos N. I. McKune A. J. Naumovski N. (2020). Anthocyanins and human health–a focus on oxidative stress, inflammation and disease. Antioxid. (Basel) 9, 366. doi: 10.3390/antiox9050366 PMC7278778 32353990

[B70] Spooner D. M. Núñez J. Trujillo G. Del Rosario H. M. Guzmán F. Ghislain M. (2007). Extensive simple sequence repeat genotyping of potato landraces supports a major reevaluation of their gene pool structure and classification. Proc. Natl. Acad. Sci. U.S.A. 104, 19398–19403. doi: 10.1073/pnas.0709796104 18042704 PMC2148301

[B71] Stushnoff C. Holm D. Thompson M. Jiang W. Thompson H. Joyce N.. (2008). Antioxidant properties of cultivars and selections from the Colorado potato breeding program. Am. J. Potato Res. 85, 267–276. doi: 10.1007/s12230-008-9032-4

[B72] Sui X. Dong X. Zhou W. (2014). Combined effect of pH and high temperature on the stability and antioxidant capacity of two anthocyanins in aqueous solution. Food Chem. 163, 163–170. doi: 10.1016/j.foodchem.2014.04.075 24912712

[B73] Thompson M. Thompson H. McGinley J. Neil E. Rush D. Holm D. (2009). Functional food characteristics of potato cultivars (Solanum tuberosum l.): Phytochemical composition and inhibition of 1-methyl-1-nitrosourea induced breast cancer in rats. J. Food Compos. Anal. 22, 571–576. doi: 10.1016/j.jfca.2008.09.002

[B74] Tillault A. S. Yevtushenko D. P. (2019). Simple sequence repeat analysis of new potato varieties developed in Alberta, Canada. Plant Direct. 3, e00140. doi: 10.1002/pld3.140 31245780 PMC6551368

[B75] Valiñas M. Lanteri M. ten Have A. Andreu A. (2017). Chlorogenic acid, anthocyanin and flavan-3-ol biosynthesis in flesh and skin of Andean potato tubers (*Solanum tuberosum* subsp. *andigena*). Food Chem. 229, 837–846. doi: 10.1016/j.foodchem.2017.02.150 28372251

[B76] Wang Y. Rashid M. Li X. Yao C. Lu L. Bai J.. (2019). Collection and evaluation of genetic diversity and population structure of potato landraces and varieties in China. Front. Plant Sci. 10, 139. doi: 10.3389/fpls.2019.00139 30846993 PMC6393402

[B77] Xu J. Zhang Y. Ren G. Yang R. Chen J. Xiang X.. (2020). Inhibitory effect of delphinidin on oxidative stress induced by H2O2 in HepG2 cells. Oxid. Med. Cell Longev. 20, 4694760. doi: 10.1155/2020/4694760 PMC7700032 33274001

[B78] Zhang H. Mittal N. Leamy L. J. Barazani O. Song B. H. (2017). Back into the wild–apply untapped genetic diversity of wild relatives for crop improvement. Evol.Appl 10, 5–24. doi: 10.1111/eva.12434 28035232 PMC5192947

[B79] Zhu F. Cai Y. Ke J. Corke H. (2010). Compositions of phenolic compounds, amino acids and reducing sugars in commercial potato varieties and their effects on acrylamide formation. J. Sci. Food Agric. 90, 2254–2262. doi: 10.1002/jsfa.4079 20629114

