# Supplementary material for: Phenolic and anthocyanin content characterization related to genetic diversity analysis of Solanum tuberosum subsp. tuberosum Chilotanum Group in southern Chile
Source: Front Plant Sci. 2023 Jan 10;13:1045894. doi: 10.3389/fpls.2022.1045894 (PMC9872146; doi:10.3389/fpls.2022.1045894)
Supplement: Supplementary file 1 [file DataSheet_1.docx]

Supplementary Material

**Phenolic and anthocyanin content characterization related to genetic diversity analysis of *Solanum tuberosum* subsp *tuberosum* Chilotanum Group in Southern Chile.**


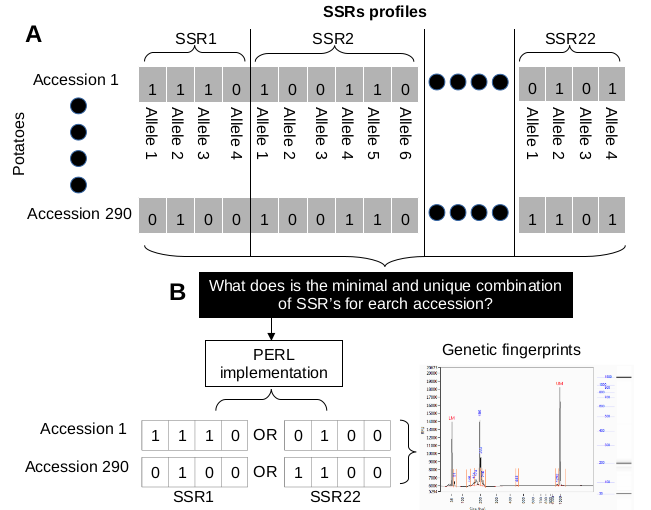


**Supplementary figure 1.** Calculation of the minimum and unique genetic fingerprint for the total number of accessions in this study. (A) PCR fragments were analyzed by capillary electrophoresis and scored as presence = 1 or absence = 0 of allele in CSV spreadsheet format representing a static binary matrix for the 290 potato accessions. (B) To calculate the minimum unique SSR combination representing a potato species (accession), a Perl implementation supported by a database design was designed to search for a unique PCR band profile for each species. A species can have multiple unique SSR representations that represent a genetic fingerprint marker with a PCR band profile that characterizes a potato species.

**Supplementary table 1.** Information about the accessions belonging to each cluster.

|  | **Identification of accessions** | **Number of accesions** |
| --- | --- | --- |
| **Cluster A** | 6,11,93,177,108,288,213,29,284,131,160,113,256,184,262,34,143,268,194,88,94,236,18,135,178,176 | 26 |
| **Cluster B** | 102,261,33,64,265,89,282,259,264,91,183,81,281,255,254,137,7,39,158,111,112,23,101,74,121,162,199,225,269,280,63,169,107,69,70,72,186,10,193,197,25,27,12,44,266,118,181,245,149,198,228,2,85,156,15,19,36,62,90,73,175,238,128,244 | 64 |
| **Cluster C** | 289,270,180,267,239,243,119,51,221,216,65,114,103,32,287,59,55,258,42,31,26,260,190,188,97,191,271,187,99,182,84,82,248,283,226,210,22,167,144,52,173,13,163 | 43 |
| **Cluster D** | 253,251,168,146,98,96,66,174,130,136,207,37,83,279,78,277,274,195,273,275,272,257,215,204,80,276,285,286,206,61,57 | 31 |
| **Cluster E** | 133,208,219,170,224,153,209,139,148,147,129,123,217,122,58,218,24,49,202,126,138,124,41,104,20,140,16,106,134,231,79,250,3 | 33 |
| **Cluster F** | 8,71,53,115,233,125,234,145,157,212,230,47,76,75,77,229,141,237,9,263,120,192,92,43,196,14,68,200,205,95,110,185,246,54,116,87,151,290,159,21,161,117,203,164,165,166,60,67,86,109,241,17,223,232 | 54 |
| **Cluster G** | 45,1,38,220,211,222,152,154,28,155,172,105,35,40,127,132,242,249,4,56,235,252,150,240,100,179,189,5,227,247,171,201,278,30,46,214,142,48,50 | 39 |
|  | **TOTAL** | **290** |

**Supplementary Table 2.** Heat map of the probability of occurrence of any of these colors in each cluster. Bolded probabilities signify a p-value < 0.05

| **Skin primary color** | |  | |  | |  | |  | |  | |  | |  |
| --- | --- | --- | --- | --- | --- | --- | --- | --- | --- | --- | --- | --- | --- | --- |
|  | **A** | **B** | | **C** | | **D** | | **E** | | **F** | | **G** | |  |
| White cream | 0,00 | 0,00 | | 0,00 | | 0,00 | | 0,00 | | 0,00 | | 0,00 | |  |
| Yellow | 0,04 | 0,13 | | 0,06 | | 0,03 | | 0,10 | | 0,06 | | 0,00 | |  |
| Orange | 0,15 | 0,10 | | 0,06 | | 0,17 | | 0,10 | | 0,20 | | 0,00 | |  |
| Brown | 0,23 | 0,22 | | 0,23 | | 0,03 | | 0,30 | | 0,24 | | 0,35 | |  |
| Pink | 0,04 | 0,12 | | 0,06 | | 0,07 | | 0,10 | | 0,04 | | 0,15 | |  |
| Red | 0,08 | 0,05 | | 0,14 | | 0,14 | | 0,13 | | 0,08 | | 0,23 | |  |
| Deep pink | 0,12 | 0,07 | | 0,09 | | 0,07 | | 0,10 | | 0,08 | | 0,04 | |  |
| Purple | 0,19 | 0,18 | | 0,14 | | 0,34 | | 0,07 | | 0,26 | | 0,19 | |  |
| Blackish | 0,15 | 0,13 | | 0,23 | | 0,14 | | 0,10 | | 0,04 | | 0,04 | |  |
| **Skin secondary color** | | |  | |  | |  | |  | |  | |  | |
|  | **A** | | **B** | | **C** | | **D** | | **E** | | **F** | | **G** | |
| White cream | 0,00 | | 0,03 | | 0,00 | | 0,00 | | 0,00 | | 0,00 | | 0,00 | |
| Yellow | 0,00 | | 0,20 | | 0,09 | | 0,09 | | 0,23 | | 0,13 | | 0,08 | |
| Orange | 0,17 | | 0,07 | | 0,00 | | 0,09 | | 0,00 | | 0,00 | | 0,08 | |
| Brown | 0,00 | | 0,07 | | 0,00 | | 0,45 | | 0,00 | | 0,09 | | 0,15 | |
| Pink | 0,17 | | 0,13 | | 0,18 | | 0,09 | | 0,08 | | 0,09 | | 0,08 | |
| Red | 0,17 | | 0,03 | | 0,27 | | 0,00 | | 0,46 | | 0,09 | | 0,31 | |
| Deep pink | **0,50** | | 0,23 | | 0,27 | | 0,09 | | 0,15 | | **0,43** | | 0,23 | |
| Purple | 0,00 | | 0,23 | | 0,18 | | 0,18 | | 0,08 | | 0,13 | | 0,08 | |
| Blackish | 0,00 | | 0,00 | | 0,00 | | 0,00 | | 0,00 | | 0,04 | | 0,00 | |
| **Flesh primary color** | |  | |  | |  | |  | |  | |  | |  |
|  | **A** | **B** | | **C** | | **D** | | **E** | | **F** | | **G** | |  |
| White | 0,00 | 0,03 | | 0,03 | | 0,00 | | 0,00 | | 0,00 | | 0,00 | |  |
| Cream | 0,44 | **0,54** | | **0,36** | | 0,44 | | 0,41 | | **0,34** | | 0,31 | |  |
| Light yellow | 0,39 | 0,21 | | 0,25 | | 0,15 | | 0,28 | | 0,21 | | **0,42** | |  |
| Yellow | 0,17 | 0,08 | | 0,19 | | 0,22 | | 0,16 | | 0,26 | | 0,23 | |  |
| Intense yellow | 0,00 | 0,00 | | 0,03 | | 0,00 | | 0,03 | | 0,05 | | 0,04 | |  |
| Red | 0,00 | 0,02 | | 0,00 | | 0,00 | | 0,00 | | 0,00 | | 0,00 | |  |
| Pink | 0,00 | 0,07 | | 0,06 | | 0,07 | | 0,00 | | 0,00 | | 0,00 | |  |
| Purple | 0,00 | 0,05 | | 0,08 | | 0,11 | | 0,13 | | 0,13 | | 0,00 | |  |
| Blackish | 0,00 | 0,00 | | 0,00 | | 0,00 | | 0,00 | | 0,00 | | 0,00 | |  |
| **Flesh secondary color** | | |  | |  | |  | |  | |  | |  | |
| **Cluster** | **A** | | **B** | | **C** | | **D** | | **E** | | **F** | | **G** | |
| White | 0,00 | | 0,00 | | 0,00 | | 0,10 | | 0,00 | | 0,14 | | 0,00 | |
| Cream | 0,00 | | 0,35 | | 0,44 | | 0,30 | | 0,30 | | **0,57** | | 0,00 | |
| Light yellow | 0,00 | | 0,12 | | 0,00 | | 0,10 | | 0,10 | | 0,00 | | 0,00 | |
| Yellow | 0,00 | | 0,00 | | 0,00 | | 0,00 | | 0,00 | | 0,00 | | 0,00 | |
| Intense yellow | 0,00 | | 0,00 | | 0,00 | | 0,00 | | 0,00 | | 0,00 | | 0,00 | |
| Red | 0,00 | | 0,00 | | 0,00 | | 0,20 | | 0,20 | | 0,00 | | **0,50** | |
| Pink | **0,75** | | 0,12 | | 0,33 | | 0,20 | | 0,30 | | 0,00 | | 0,17 | |
| Purple | 0,25 | | 0,41 | | 0,22 | | 0,10 | | 0,10 | | 0,29 | | 0,33 | |
| Blackish | 0,00 | | 0,00 | | 0,00 | | 0,00 | | 0,00 | | 0,00 | | 0,00 | |

| Color range | 0 | 0,2 | 0,4 | 0,6 | 0,8 | 1 |
| --- | --- | --- | --- | --- | --- | --- |
| Significance | p<0.05 |  |  |  |  |  |
